# 2-Bromo-4-phenyl-1,3-thia­zole

**DOI:** 10.1107/S160053681400066X

**Published:** 2014-01-15

**Authors:** Alexander S. Bunev, Yana I. Rudakova, Vladimir E. Statsyuk, Gennady I. Ostapenko, Victor N. Khrustalev

**Affiliations:** a Department of Chemistry and Chemical Technology, Togliatti State University, 14 Belorusskaya St, Togliatti 445667, Russian Federation; b X-Ray Structural Centre, A.N. Nesmeyanov Institute of Organoelement Compounds, Russian Academy of Sciences, 28 Vavilov Street, B-334, Moscow 119991, Russian Federation

## Abstract

In the title mol­ecule, C_9_H_6_BrNS, the planes of the 2-bromo-1,3-thia­zole and phenyl rings are inclined at 7.45 (10)° with respect to each other. In the crystal, mol­ecules related by a centre of symmetry are held together *via* π–π inter­actions, with a short distance of 3.815 (2) Å between the centroids of the five- and six-membered rings. The crystal packing exhibits short inter­molecular S⋯Br contacts of 3.5402 (6) Å.

## Related literature   

For syntheses and properties of compounds containing a thia­zole fragment, see: Kelly & Lang (1995 ▶); Nicolaou *et al.* (1999 ▶); Cosford *et al.* (2003 ▶); Fyfe *et al.* (2004 ▶); Hamill *et al.* (2005 ▶). For the crystal structures of related compounds, see: Abbenante *et al.* (1996 ▶); Zhao *et al.* (2011 ▶); Ghabbour, Chia *et al.* (2012 ▶); Ghabbour, Kadi *et al.* (2012 ▶).
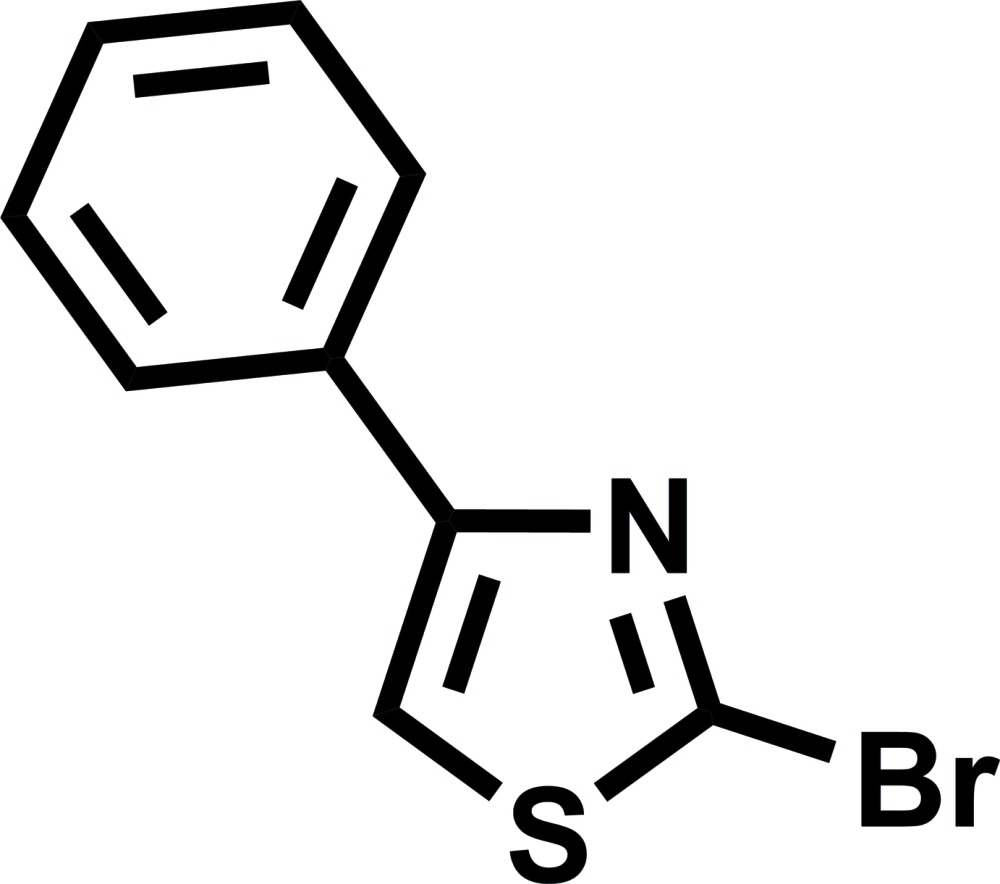


## Experimental   

### 

#### Crystal data  

C_9_H_6_BrNS
*M* *_r_* = 240.12
Monoclinic, 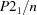

*a* = 5.8934 (3) Å
*b* = 10.6591 (6) Å
*c* = 13.8697 (7) Å
β = 90.812 (1)°
*V* = 871.18 (8) Å^3^
*Z* = 4
Mo *K*α radiation
μ = 4.89 mm^−1^
*T* = 120 K
0.15 × 0.12 × 0.12 mm

#### Data collection  

Bruker APEXII CCD diffractometer
Absorption correction: multi-scan (*SADABS*; Bruker, 2003 ▶) *T*_min_ = 0.527, *T*_max_ = 0.591
12144 measured reflections
2780 independent reflections
2258 reflections with *I* > 2σ(*I*)
*R*_int_ = 0.045

#### Refinement  

*R*[*F* ^2^ > 2σ(*F* ^2^)] = 0.029
*wR*(*F* ^2^) = 0.068
*S* = 1.03
2780 reflections
109 parameters
H-atom parameters constrained
Δρ_max_ = 0.40 e Å^−3^
Δρ_min_ = −0.51 e Å^−3^

### 

Data collection: *APEX2* (Bruker, 2005 ▶); cell refinement: *SAINT* (Bruker, 2001 ▶); data reduction: *SAINT*; program(s) used to solve structure: *SHELXTL* (Sheldrick, 2008 ▶); program(s) used to refine structure: *SHELXTL*; molecular graphics: *SHELXTL*; software used to prepare material for publication: *SHELXTL*.

## Supplementary Material

Crystal structure: contains datablock(s) global, I. DOI: 10.1107/S160053681400066X/cv5440sup1.cif

Structure factors: contains datablock(s) I. DOI: 10.1107/S160053681400066X/cv5440Isup2.hkl

Supporting information file. DOI: 10.1107/S160053681400066X/cv5440Isup3.cml

CCDC reference:

Additional supporting information:  crystallographic information; 3D view; checkCIF report

## Figures and Tables

**Fig. 1.**
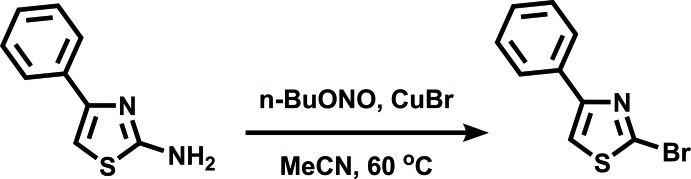
Synthesis of 2–bromo–4–phenylthiazole.

**Fig. 2.**
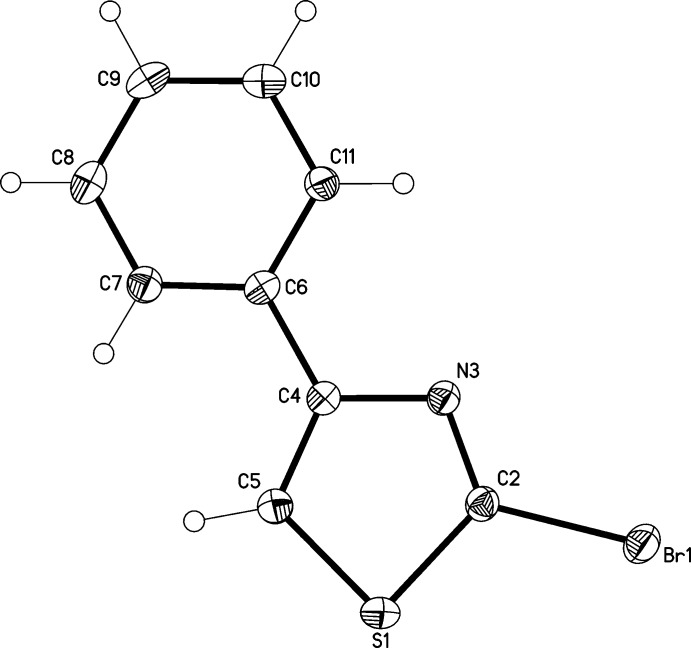
Molecular structure of I. Displacement ellipsoids are presented at the 50% probability level. H atoms are depicted as small spheres of arbitrary radius.

**Fig. 3.**
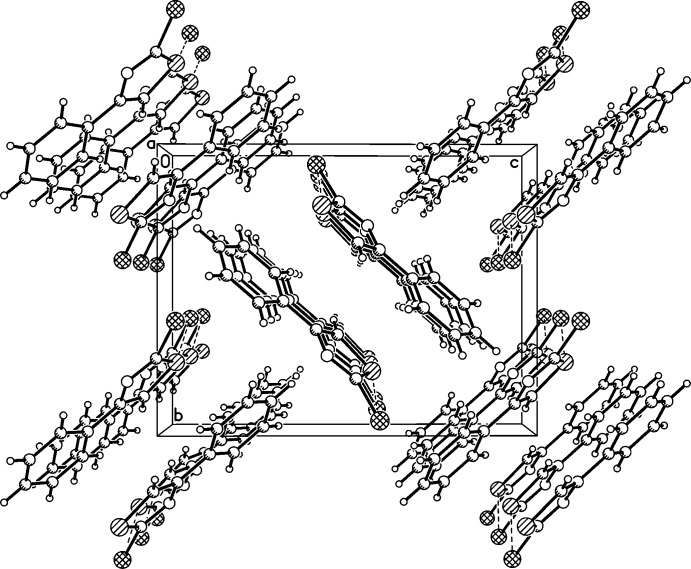
The crystal packing of I viewed along the a axis. Dashed lines indicate the intermolecular secondary S···Br interactions.

## supplementary crystallographic information

### 1. Comment

1,3–Thiazole rings appear in many compounds that exhibit important biological and pharmacological activities. For example, these rings feature in all the potent epothilones (Nicolaou *et al.*, 1999) used aganist multidrug–resistant tumor cell lines. They are also found among pharmaceuticals used for the treatment of type 2 diabetes (Fyfe *et al.*, 2004), antibiotic-like compounds (Kelly *et al.*, 1995), and metabotropic glutamate receptor subtype (mGluR5) antagonists (Cosford *et al.*, 2003; Hamill *et al.*, 2005). Herewith we present the title compound (I) prepared by the reaction of 2–amino–4–phenylthiazole with *n*-butyl nintrine and CuBr (Figure 1).

In I (Fig. 2), the bond lengths and angles are in a good agreement with those found in the related compounds (Abbenante *et al.*, 1996; Zhao *et al.*, 2011; Ghabbour, Chia *et al.*, 2012; Ghabbour, Kadi *et al.*, 2012). The 2-bromo-1,3-thiazole mean plane and phenyl ring are twisted by 7.45 (10)°.

In the crystal, the molecules related by center of symmetry held together *via*π···π interactions proved by short *Cg*^5^···*Cg*^6i^ distance of 3.815 (2) Å between the centroids of five-membered (*Cg*^5^) and six-membered (*Cg*^6^) rings [symmetry code: (i) –*x*, 1–*y*, 1–*z*]. The crystal packing exhibits short intermolecular S···Br^ii^ contacts of 3.5402 (6) Å (Figure 3) [symmetry code: (ii) -1 + *x*, *y*, *z*].

### 2. Experimental

The 4–phenyl–2–aminothiazole (8.1 g, 46.9 mmol) and CuBr (10.7 g, 74.6 mmol) were dissolved in acetonitrile at room temperature. *n*-Butyl nitrite (8.7 ml, 7.69 g, 74.6 mmol) was added with stirring, and the solution was heated to 333 K. The reaction completed after 15 min. The reaction mixture was then evaporated to dryness in *vacuo*. The residue was dissolved in ethyl acetate (50 ml) and washed with ammonia solution (0.1 *M*, 2 × 50). The organic layer was dried over MgSO_4_ and evaporated to dryness in *vacuo*. The residue was purified by chromatography on silica gel (heptane–ethylacetate; 70:3, *v*/*v*). The residue crystallized from 5% soluition in heptane. Yield is 53%. The single-crystal of the product I was obtained by slow crystallization from hexane. *M*.p. = 327–328 K. IR (KBr), ν/cm^-1^: 3098, 3063, 1476, 1420, 1263, 1070, 1010, 836, 730, 689. ^1^H NMR (500 MHz, DMSO-*d_6_*, 304 K): 7.40–6.37 (m, 1H, Ph), 7.46 (t, 2H, *J* = 7.63, Ph), 7.92 (d, 2H, *J* = 7.32, Ph), 8.16 (s, 1H, thiazole). Anal. Calcd for C_9_H_6_BrNS: C, 45.02; H, 2.52. Found: C, 45.09; H, 2.57.

### 3. Refinement

All hydrogen atoms were placed in the calculated positions [C—H = 0.95 Å] and refined in the riding model, with *U*_iso_(H) = 1.2*U*_eq_(C)].

### Crystal data

**Table:**

C_9_H_6_BrNS	*F*(000) = 472
*M**_r_* = 240.12	*D*_x_ = 1.831 Mg m^−^^3^
Monoclinic, *P*2_1_/*n*	Mo *K*α radiation, λ = 0.71073 Å
Hall symbol: -P 2yn	Cell parameters from 3185 reflections
*a* = 5.8934 (3) Å	θ = 2.4–29.5°
*b* = 10.6591 (6) Å	µ = 4.89 mm^−^^1^
*c* = 13.8697 (7) Å	*T* = 120 K
β = 90.812 (1)°	Prism, yellow
*V* = 871.18 (8) Å^3^	0.15 × 0.12 × 0.12 mm
*Z* = 4	

### Data collection

**Table:**

Bruker APEXII CCD diffractometer	2780 independent reflections
Radiation source: fine–focus sealed tube	2258 reflections with *I* > 2σ(*I*)
Graphite monochromator	*R*_int_ = 0.045
φ and ω scans	θ_max_ = 31.0°, θ_min_ = 2.4°
Absorption correction: multi-scan (*SADABS*; Bruker, 2003)	*h* = −8→8
*T*_min_ = 0.527, *T*_max_ = 0.591	*k* = −15→15
12144 measured reflections	*l* = −20→19

### Refinement

**Table:**

Refinement on *F*^2^	Primary atom site location: structure-invariant direct methods
Least-squares matrix: full	Secondary atom site location: difference Fourier map
*R*[*F*^2^ > 2σ(*F*^2^)] = 0.029	Hydrogen site location: inferred from neighbouring sites
*wR*(*F*^2^) = 0.068	H-atom parameters constrained
*S* = 1.03	*w* = 1/[σ^2^(*F*_o_^2^) + (0.0323*P*)^2^ + 0.1245*P*] where *P* = (*F*_o_^2^ + 2*F*_c_^2^)/3
2780 reflections	(Δ/σ)_max_ = 0.002
109 parameters	Δρ_max_ = 0.40 e Å^−^^3^
0 restraints	Δρ_min_ = −0.51 e Å^−^^3^

### Special details

**Table:**

Geometry. All e.s.d.'s (except the e.s.d. in the dihedral angle between two l.s. planes) are estimated using the full covariance matrix. The cell e.s.d.'s are taken into account individually in the estimation of e.s.d.'s in distances, angles and torsion angles; correlations between e.s.d.'s in cell parameters are only used when they are defined by crystal symmetry. An approximate (isotropic) treatment of cell e.s.d.'s is used for estimating e.s.d.'s involving l.s. planes.
Refinement. Refinement of *F*^2^ against ALL reflections. The weighted *R*-factor *wR* and goodness of fit *S* are based on *F*^2^, conventional *R*-factors *R* are based on *F*, with *F* set to zero for negative *F*^2^. The threshold expression of *F*^2^ > σ(*F*^2^) is used only for calculating *R*-factors(gt) *etc*. and is not relevant to the choice of reflections for refinement. *R*-factors based on *F*^2^ are statistically about twice as large as those based on *F*, and *R*- factors based on ALL data will be even larger.

### Fractional atomic coordinates and isotropic or equivalent isotropic displacement parameters (Å^2^)

**Table:**

	*x*	*y*	*z*	*U*_iso_*/*U*_eq_	
Br1	0.21824 (3)	0.90020 (2)	0.576233 (15)	0.02238 (7)	
S1	−0.24024 (8)	0.75866 (5)	0.55984 (4)	0.01975 (11)	
C2	0.0322 (3)	0.77659 (19)	0.52049 (14)	0.0165 (4)	
N3	0.0960 (3)	0.70330 (16)	0.45150 (11)	0.0161 (3)	
C4	−0.0825 (3)	0.62475 (17)	0.42443 (14)	0.0144 (4)	
C5	−0.2768 (3)	0.64278 (19)	0.47549 (14)	0.0178 (4)	
H5	−0.4135	0.5972	0.4655	0.021*	
C6	−0.0500 (3)	0.53485 (18)	0.34463 (14)	0.0151 (4)	
C7	−0.2155 (3)	0.4453 (2)	0.32167 (14)	0.0184 (4)	
H7	−0.3508	0.4419	0.3580	0.022*	
C8	−0.1849 (4)	0.3613 (2)	0.24652 (14)	0.0214 (4)	
H8	−0.2988	0.3009	0.2318	0.026*	
C9	0.0134 (4)	0.3655 (2)	0.19254 (15)	0.0213 (4)	
H9	0.0354	0.3080	0.1411	0.026*	
C10	0.1778 (4)	0.4544 (2)	0.21484 (15)	0.0208 (4)	
H10	0.3130	0.4576	0.1784	0.025*	
C11	0.1471 (3)	0.53900 (19)	0.28978 (14)	0.0174 (4)	
H11	0.2605	0.5999	0.3038	0.021*	

### Atomic displacement parameters (Å^2^)

**Table:**

	*U*^11^	*U*^22^	*U*^33^	*U*^12^	*U*^13^	*U*^23^
Br1	0.02065 (11)	0.02080 (11)	0.02561 (12)	−0.00021 (8)	−0.00253 (8)	−0.00725 (8)
S1	0.0181 (2)	0.0210 (3)	0.0202 (2)	0.00233 (19)	0.00358 (19)	−0.00361 (19)
C2	0.0156 (9)	0.0158 (9)	0.0181 (9)	0.0006 (7)	−0.0012 (7)	−0.0013 (7)
N3	0.0167 (8)	0.0160 (8)	0.0155 (8)	−0.0008 (6)	−0.0005 (6)	−0.0006 (6)
C4	0.0162 (9)	0.0129 (9)	0.0142 (8)	0.0009 (7)	−0.0008 (7)	0.0015 (7)
C5	0.0173 (9)	0.0173 (9)	0.0190 (10)	0.0002 (7)	0.0009 (7)	−0.0011 (8)
C6	0.0187 (9)	0.0137 (9)	0.0129 (8)	0.0020 (7)	−0.0013 (7)	0.0015 (7)
C7	0.0194 (9)	0.0182 (9)	0.0176 (9)	−0.0013 (8)	0.0002 (7)	0.0010 (8)
C8	0.0264 (10)	0.0180 (10)	0.0198 (10)	−0.0019 (8)	−0.0054 (8)	−0.0004 (8)
C9	0.0307 (11)	0.0180 (10)	0.0153 (9)	0.0045 (8)	−0.0019 (8)	−0.0026 (7)
C10	0.0224 (10)	0.0225 (10)	0.0175 (9)	0.0041 (8)	0.0028 (8)	0.0006 (8)
C11	0.0184 (9)	0.0165 (9)	0.0172 (9)	−0.0013 (7)	0.0019 (7)	−0.0004 (7)

### Geometric parameters (Å, º)

**Table:**

Br1—C2	1.874 (2)	C7—C8	1.388 (3)
S1—C5	1.713 (2)	C7—H7	0.9500
S1—C2	1.714 (2)	C8—C9	1.398 (3)
C2—N3	1.295 (2)	C8—H8	0.9500
N3—C4	1.392 (2)	C9—C10	1.388 (3)
C4—C5	1.368 (3)	C9—H9	0.9500
C4—C6	1.478 (3)	C10—C11	1.390 (3)
C5—H5	0.9500	C10—H10	0.9500
C6—C11	1.398 (3)	C11—H11	0.9500
C6—C7	1.398 (3)		
			
C5—S1—C2	88.40 (10)	C8—C7—H7	119.5
N3—C2—S1	116.81 (15)	C6—C7—H7	119.5
N3—C2—Br1	123.68 (15)	C7—C8—C9	120.0 (2)
S1—C2—Br1	119.49 (11)	C7—C8—H8	120.0
C2—N3—C4	109.64 (17)	C9—C8—H8	120.0
C5—C4—N3	114.24 (17)	C10—C9—C8	119.28 (19)
C5—C4—C6	126.63 (18)	C10—C9—H9	120.4
N3—C4—C6	119.11 (17)	C8—C9—H9	120.4
C4—C5—S1	110.91 (15)	C9—C10—C11	120.80 (19)
C4—C5—H5	124.5	C9—C10—H10	119.6
S1—C5—H5	124.5	C11—C10—H10	119.6
C11—C6—C7	118.68 (18)	C10—C11—C6	120.30 (19)
C11—C6—C4	120.27 (17)	C10—C11—H11	119.9
C7—C6—C4	121.05 (17)	C6—C11—H11	119.9
C8—C7—C6	120.92 (19)		
			
C5—S1—C2—N3	−0.65 (17)	C5—C4—C6—C7	−8.5 (3)
C5—S1—C2—Br1	178.28 (13)	N3—C4—C6—C7	173.20 (18)
S1—C2—N3—C4	0.5 (2)	C11—C6—C7—C8	0.5 (3)
Br1—C2—N3—C4	−178.35 (13)	C4—C6—C7—C8	179.78 (19)
C2—N3—C4—C5	−0.1 (2)	C6—C7—C8—C9	0.0 (3)
C2—N3—C4—C6	178.41 (17)	C7—C8—C9—C10	−0.2 (3)
N3—C4—C5—S1	−0.4 (2)	C8—C9—C10—C11	−0.1 (3)
C6—C4—C5—S1	−178.74 (16)	C9—C10—C11—C6	0.6 (3)
C2—S1—C5—C4	0.56 (16)	C7—C6—C11—C10	−0.8 (3)
C5—C4—C6—C11	170.74 (19)	C4—C6—C11—C10	179.93 (18)
N3—C4—C6—C11	−7.5 (3)		
